# Breast tumor-on-chip models: From disease modeling to personalized drug screening

**DOI:** 10.1016/j.jconrel.2020.12.057

**Published:** 2021-03-10

**Authors:** Bano Subia, Ujjwal Ranjan Dahiya, Sarita Mishra, Jessica Ayache, Guilhem Velve Casquillas, David Caballero, Rui L. Reis, Subhas C. Kundu

**Affiliations:** a Elvesys Microfluidics Innovation Centre, Paris 75011, France.; b CSIR-Institute of Genomics and Integrative Biology, New Delhi 110025, India.; c 3B's Research Group, I3Bs−Institute on Biomaterials, Biodegradables and Biomimetics, Headquarters of the European Institute of Excellence on Tissue Engineering and Regenerative Medicine, University of Minho, AvePark, Barco, Guimarãaes 4805-017, Portugal; d ICVS/3B's – PT Government Associate Laboratory, 4805-017, Braga/Guimarães, Portugal

**Keywords:** Breast cancer, Microfluidics, Tumor-on-chip, Drug screening, Industrial applications, *2D*, 2 dimensional, *3D*, 3 dimensional, *BRCA1*, Breast cancer associated gene 1, *BRCA2*, Breast cancer associated gene 2, *BME2rgf*, Cultrex® Basement membrane extract, *CAAs*, Carcinoma associated adipocytes, *CAFs*, Carcinoma associated fibroblasts, *CTCs*, Circulating tumor cells, *DCIS*, Ductal carcinoma *in situ*, *ECM*, Extracellular matrix, *EGF*, Epidermal growth factor, *EGFR*, Epidermal growth factor receptor, *ER*, Estrogen receptor, *ERβ*, Estrogen receptor β, *GPNMB*, glycoprotein non-metastatic B, *hBM-MSCs*, Human bone marrow-derived mesenchymal stem cells, *HER2*, Human epidermal growth factor receptor 2, *HIF-1*, Hypoxia-inducible factor-1, *HUVEC*, Human umbilical vein endothelial cell, *IF*, Interstitial fluid, *IL-8*, Interleukin-8, *MMPs*, Matrix metalloproteinases, *MOC*, Multiorgan-on-chip, *PDGF*, Platelet derive growth factors, *PDX*, Patient-derived xenografts, *PK/PD*, Pharmacokinetics and pharmacodynamics, *PR*, Progesterone receptors, *TIF*, Tumor interstitial fluid, *TIME*, Telomerase-immortalized-human microvascular endothelial, *TME*, Tumor microenvironment, *VEGF*, Vascular endothelial growth factor

## Abstract

Breast cancer is one of the leading causes of mortality worldwide being the most common cancer among women. Despite the significant progress obtained during the past years in the understanding of breast cancer pathophysiology, women continue to die from it. Novel tools and technologies are needed to develop better diagnostic and therapeutic approaches, and to better understand the molecular and cellular players involved in the progression of this disease. Typical methods employed by the pharmaceutical industry and laboratories to investigate breast cancer etiology and evaluate the efficiency of new therapeutic compounds are still based on traditional tissue culture flasks and animal models, which have certain limitations. Recently, tumor-on-chip technology emerged as a new generation of *in vitro* disease model to investigate the physiopathology of tumors and predict the efficiency of drugs in a native-like microenvironment. These microfluidic systems reproduce the functional units and composition of human organs and tissues, and importantly, the rheological properties of the native scenario, enabling precise control over fluid flow or local gradients. Herein, we review the most recent works related to breast tumor-on-chip for disease modeling and drug screening applications. Finally, we critically discuss the future applications of this emerging technology in breast cancer therapeutics and drug development.

## Highlights

• Breast cancer is one of the leading causes of mortality among women.
• Microfluidics can address the challenges related to breast cancer progression.
• Breast cancer physiopathology can be replicated using tumor-on-chip technology.
• Tumor biomarkers can be precisely identified and analyzed.
• Drug screening and discovery can be accelerated with microfluidics.

## Graphical abstract

**Figure:**
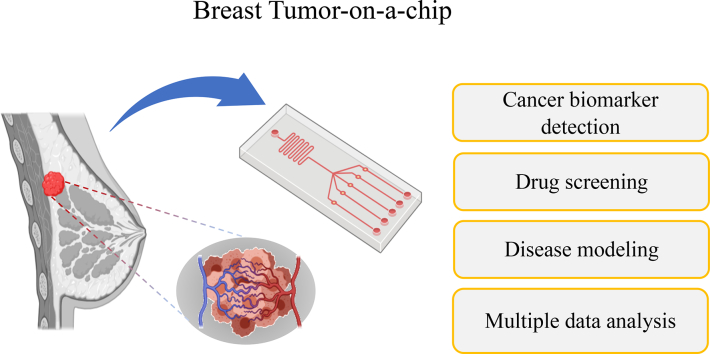

## 1 Introduction

Breast cancer is one of the primary causes of death in women worldwide, with around 20% of morbidities associated to it [1]. Breast cancer development, like any other neoplasm, is a complex multistep process with a high level of molecular and morphological heterogeneities [2]. Understanding the breast cancer progression and the underlying heterogeneity are important to address the challenges related to the mechanisms of tumor invasion, metastasis and drug action [3]. Traditionally, the efforts in this direction have mainly focused on using conventional two- (2D) and three-dimensional (3D) cell culture systems and animal models. The formers, which include tissue culture flasks, transwell plates, scaffolds, or spheroids, can mimic some of the events occurring during tumor progression. In particular, 3D models can recapitulate the native cell-cell and cell-matrix interactions, proliferation, migration and drug responses [4]. Even though these models are less expensive and provide a higher level of reproducibility, they still display serious limitations. They are incapable to reproduce the physicochemical properties of the native tumor microenvironment (TME) and lack fluid flow, tissue deformation, and shear stress, which play major role in cancer cell invasion [5]. In contrast, animal models can reproduce better biological and structural complexities of the native scenario providing essential information about the *in vivo* tumor physiology. However, they are not predictive of the actual effect of drugs in humans; they are also ethically controversial and highly expensive [6]. Lately, more sophisticated 3D *in vitro* model based on organoid technology emerged as biomimetic platforms for drug discovery. Organoids involve the culture of healthy or cancerous epithelial stem cells isolated from the donor [7]. The organoid culture resembles the *in vivo* scenario along with showing genotype-phenotype correlation [8]. However, despite their advanced capabilities for being employed as predictive and screening platforms, organoids display certain limitations, such as the incapability of a direct experimental access to the epithelial lumen, which limits their applicability [9].

During the last decade, the combination of nanotechnology, biomaterials, tissue engineering, oncology, and pharmacology have resulted in the development of organ-on-chip systems. These are microfluidics-based *in vitro* models, which are considered a promising alternative to reproduce the functional units of tissues or organs. When combined with cancer cells, these models are denoted as cancer- or tumor-on-a-chip and can be employed to investigate the mechanistic determinants of cancer metastasis or the response of the tumor to drugs within a realistic microenvironment. This approach allows a tight control on the scaling properties of the different tissues, fluid flow, shear stress, medium and gas supply, biochemical gradient formation, or cell co-culture, among other parameters. It can also help in mimicking the physiological environment of human organs, such as cell patterning, boundaries, or tissue-organ interactions [7,8].

During the last few years, different studies have reported the use of tumor-on-chip models for a diverse variety of applications. Indeed, there is a vast literature on the topic and several recent reviews are widely available [6,9]. To the best of our knowledge, no reviews on organ-on-a-chip models exclusively focusing on breast cancer have been reported despite its tremendous clinical and social impact. Therefore, the aim of this review is to focus on putative applications of breast tumor-on-chip models for mechanistic studies and drug screening. We also describe the use of organ-on-chip systems to unravel the factors initiating breast cancer growth and progression with a special focus on the contribution of the TME. Next, we critically report on how organ-on-chip technology can contribute in the future of breast cancer research by the early-stage detection of predictive biomarkers and in the development of personalized treatments. Finally, relevant clinical and industrial applications of breast tumor-on-chip models are discussed and a consolidated future perspective based on the current understanding is presented.

## 2 Breast cancer physiology and underlying factors

Human breast physiology comprises parenchyma and stromal elements [10]. Parenchyma consist of lobes (12–20 in healthy female) and ducts. Adipose tissue fills the space between lobes and ducts, and the pectoral muscles supports breast tissue, located under the breast [11]. Stromal tissue consists of adipose and other connective tissue, which provide an environment for the development of breast parenchyma. Each lobe bears smaller lobules within it, and a tiny bulb-like structure responsible for milk production is present at the end of each lobe [10]. These structures are linked together through a small duct carrying milk. Breast cancer histology can be categorized into invasive and *in situ* carcinoma, which can further be segregated into ductal or lobular carcinoma [12]. Out of these, ductal carcinoma represents 50–75% of the total patients and is characterized by its initiation in the milk ducts and limited growth. Invasive lobular carcinoma represents 5–15% of patients. It is the next most common breast cancer type and is found in breast lobules and tissue [12]. The remaining patients are categorized into mixed ductal/lobular carcinomas or to other rarer histology [13]. Based on the underlying molecular markers, multiple types of breast cancer are identified, namely ER (estrogen receptor), PR (progesterone receptor) and HER2 (epidermal growth factor receptor 2) [14].

**Fig. 1:**
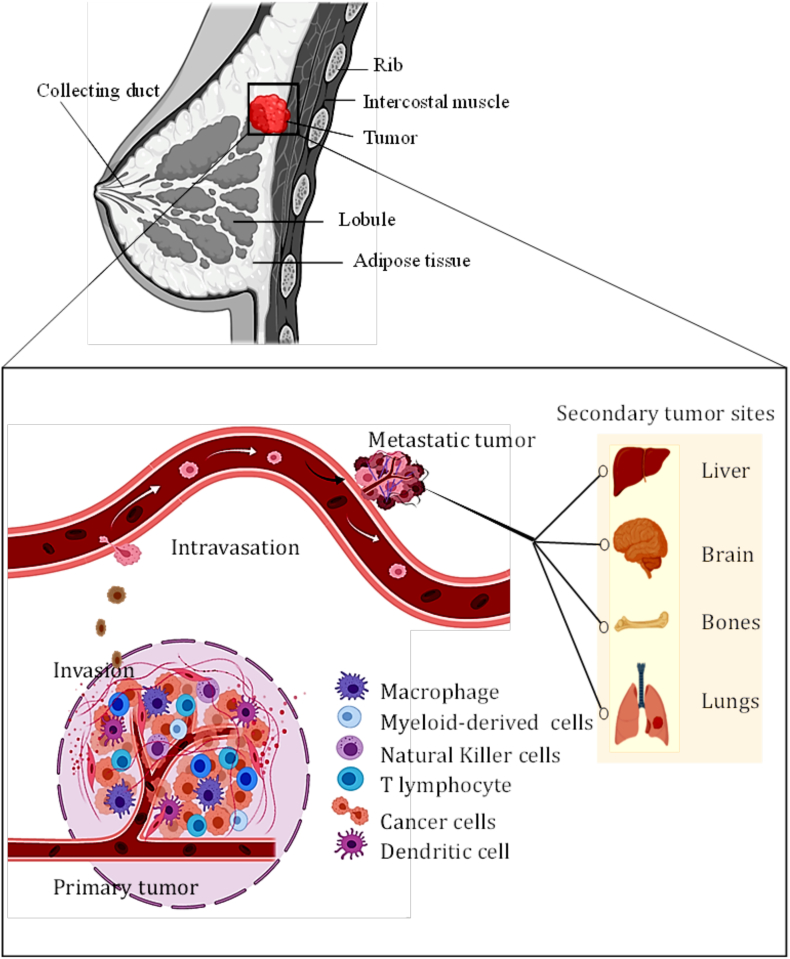
The tumor microenvironment (TME) and the cascade of breast cancer metastasis. Tumor dissemination is initiated by the uncontrolled growth of the tumor and the formation of angiogenesis, a process where new blood vessels are formed from the preexisting ones. These vessels are employed to provide nutrients and oxygen to the tumor. Next, metastatic cancer cells invade the surrounding TME and migrate directionally towards the microvasculature to invade it in a process known as *intravasation*. Then, these tumor cells travel through the blood vessels as circulating tumor cells (CTCs) to invade distant organs. Many of these CTCs are destroyed or damaged during the circulation due to their inability to transit through the capillaries. A few undamaged cells may extravasate and invade the parenchyma of a foreign tissue (*e.g.,* liver, brain, bone, or lung). At the invading stage, cancer cells start proliferating forming a secondary tumor site. Therein, multiple immune cells, such as macrophages, natural killer cells, T lymphocytes and dendritic cells, reside in the tumor niche (Created using Biorender.com).

Breast tumor begins with epithelial hyperactive proliferation and progresses to *in situ*, invasive, and metastatic carcinomas through defined stages [15]. Ductal carcinoma *in situ* (DCIS) lesions contain proliferating neoplastic cells surrounded by myoepithelial cells and an intact basement membrane. Solid evidences suggest that the DCIS may be the precursor of invasive ductal carcinoma (IDC) [13,16]. Breast tumor metastasis refers to the phenomenon when tumor cells invade and colonize distant sites that are far away from the primary tumor site. The process involves extravasation and angiogenesis at the new metastatic site, and down regulation of adhesion molecules causing the intravasation and invasion into the surrounding stroma (Fig. 1). Breast cancer development depends upon multiple factors, such as patient age and lifestyle (*e.g.*, obesity, addiction to alcohol or tobacco and others), genetic predisposition (BRCA1 or BRCA2 mutations), exposure to radiation, breast density, hyperlipidemia, or use of hormonal therapy [17]. Ovarian hormones estrogen, progesterone and prolactin are found to have important role in mammary carcinogenesis [18]. Reproductive factors like menarche and menopause in woman lead to increased exposure of breast tissue to progesterone and estrogen. Several evidences also suggest an association of full-term pregnancy and breast-feeding at an early age reduces the risk of breast cancer. The older age pregnancy shows association with luminal subtype of breast cancer [19]. Multiple studies indicate the association between non-alcoholic fatty liver disease and breast cancer occurrence worldwide [20]. Genetic predisposition manifested in terms of mutation in BRCA1 or BRCA2 genes or increased breast density, is found to increase the risk by 32% and 47% [21]. Overexpression of estrogen receptor β (ERβ) and epidermal growth factor receptors (EGFR) are also reported to play an important role in tumor progression [22]. The immune system in the progression of breast cancer is found to have an important role [23,24].

## 3 Characteristics and role of the breast tumor microenvironment (TME)

Solid tumors often resemble the structural and cellular heterogeneity of a healthy organ, comprising specialized cells performing different roles and with a sustained flow of blood [25]. Heterogeneity refers to the sub-populations of cancer cells, and it can broadly be categorized as inter-tumoral and intra-tumoral. Inter-tumor heterogeneity refers to the variability between the tumors (same or different tissue type, from different individuals with the same type of cancer) and it can be observed in circulating tumor cells cohort. Intra-tumor variability refers to the differences in cells within tumor [26]. The underlying cause of breast cancer heterogeneity is the coordination between the tumor cells and associated connective tissue cells (stromal cells) [27]. These heterogeneities play a critical role in the development of the breast TME. This in turn makes it challenging to explore the mechanism of tumor progression and potential therapeutic target. The TME is also associated with tumor metastasis and provides resistance to anti-cancer therapy [28,29]. It is characterized by various interactions between a heterogeneous population of neoplastic and stromal cells, such as fibroblasts, immune cells, and adipocytes cells, with the extracellular matrix (ECM), signaling molecules, and the vascular network. The TME plays a critical role in the successive progression of more stubborn and advanced malignancies. This is the prime contributing factor for rendering heterogeneity to tumor architecture and significantly promotes its growth [25].

The tumor growth and metastasis are an overall outcome of the interactions between the tumor and the cellular, biochemical and mechanical cues, as well as with the ECM [30]. The ECM is composed of proteins, polysaccharides and proteoglycans, and provides structural support and functional properties to the cellular system [31]. The ECM of the breast tumor niche exhibits distinct changes in composition, topography and collagen amount, leading to altered properties. Many ECM proteins, such a fibronectin, fibrillar collagens, and proteoglycans are found to be induced in breast cancer, showing association with promotion of stem/progenitor signaling and metastatic growth [32]. Also, an increased deposition of collagen I, II, III, V and IX are reported during breast tumor formation [33]. This leads to a stiffer ECM and tumorigenic environment causing enhanced cellular growth, stiffening, and compromised integrity of cell-cell junctions. Induction of multiple ECM remodeling enzyme leads to change in biochemical properties and matrix structure. This in turn results into disorganized, non-polarized and invasive colonies of cells having less cell-cell junction proteins [35].

### 3.1 Hypoxia and angiogenesis

The underlying biochemical changes in the TME include the formation of an oxygen gradient and metabolic alterations [34,35]. Tumor cells within the niche are exposed to three different oxygen conditions, namely normoxic (around functional blood vessels), hypoxic (peri-necrotic region) and necrotic (foci surrounded by hypoxic area) regions [36]. Hypoxia is one of the most significant trademark of breast cancer and affects several tumor properties by regulating hypoxia induced factors (HIFs) [37]. An important chemical change in tumor niche is the alteration in the levels of HIF-1. These are transcription factors, which respond to oxygen levels in the cells. Normally in healthy cells, HIF-1 is constitutively present and shows rapid degradation by the von-Hippel-Lindau tumor suppressor protein [38]. HIF-1α is one of the factors secreted by tumor cells, which help to the tumor cell expansion. This stimulates the growth of certain cancers, including triple-negative breast cancer [39]. It triggers vascularization (angiogenesis) by upregulation of VEGF expression and also alters the expression of proteins to change the metabolism from oxidative to glycolysis [[40], [41], [42]]. In healthy cells, HIF-1α is rapidly degraded, while in tumor cells, intracellular ascorbate [43] and glutamate levels [44] can affect these pathways resulting in HIF-1α accumulation. Hypoxia in tumor niche is also responsible for the formation of invadopodia, actin-based membrane protrusions that degrade the ECM initiating tumor cell invasion [3]. The role of hypoxia in cancer invasion is well established but its overall contribution to TME formation is yet not clear. This includes spatiotemporal organization of not only tumor cells but also endothelial, cancer-associated fibroblasts (CAFs), and immune cells, driving ECM remodeling. Angiogenesis is simultaneously induced in the tumor niche through the expression of oncogenes, such as Ras or Myc, resulting in neovascularization and making the tumor environment more complex by giving rise to different subpopulation of cancer cells. Subsequently, the number of tumor-associated macrophages (TAMs) increases up to 50% of the total tumor mass as the VEGF acts as a chemoattractant [45].

### 3.2 Metabolic reprogramming

A critical phenomenon during tumor growth is the so-called Warburg effect, wherein the cancer cells produce energy through the aerobic glycolysis rather than ATP utilization through mitochondrial oxidative phosphorylation [46]. The pyruvate generated from glycolysis is not converted to acetyl-CoA and is accumulated in the form of lactate [47]. This lactate bearing core necrotic niche exhibits high acidity along with low oxygen and promotes tumor survival and metabolic resistance to therapeutics [48]. The monocarboxylate transporters and ion pumps release lactate and H+ ions causing extracellular acidification [49,50]. The resulting acidic compartment generated through metabolic shift also promotes tumor migration by degrading E-cadherin and disrupting adherence junctions *via* Src activation [51]. The increased lactate level is reported to be a source of nutrients for tumor cells [44], inducing VEGF production [52], and immune cell evasion [53]. Technical challenges related to the isolation of tumor interstitial fluid, which involves surgical intervention, hinder the study of such critical metabolite [54,55].

### 3.3 Mechanical cues

The interstitial space generally refers to the space between the supportive and connective tissues. This consists of two major phases: interstitial fluid (IF) and the ECM [56]. IF is driven by the hydrostatic and osmotic pressure differences among the venous, arterial and lymphatic vessels [57]. It influences the cellular function through imparting shear stress by mechano-transduction [58]. This fluid can also influence the transport of nutrients and waste through extracellular gradient of soluble signaling factors, which can circuitously influence the cellular processes [59]. IF flow is generally more elevated in the tumoral tissue than in its healthy counterpart, most likely due to the abnormal tumor vasculature, unregulated vascular permeability, tumor-associated lymph-angiogenesis, and abnormal tumor stroma. It is denoted as tumor interstitial fluid (TIF), and acts as an important mechanical force. This modulates cellular behavior, cell flow, and overall tumor progression [54]. In the tumor tissue, the range of TIF pressure can reach up to 20–50 mmHg in comparison to −8 to 6 mmHg found in normal tissue [60]. This allows the outward flow of liquid from the tumor core and prevent the inward transport of molecules. High TIF pressure can cause collagen fiber alignment and fibroblast cells contraction leading to tumor stiffening. This also results into an increased tumor invasiveness as the cells can easily migrate through the aligned fibers [61]. Apart from the transport of nutrients and waste products, IF is also responsible for transporting pro-inflammatory and proangiogenic factors to the distant organs [62]. In this way, IF affects the cellular proliferation and tumor invasion potential. Therefore, investigating the role of TIF in tumor progression is crucial to understand the etiology of the disease and to develop better treatments.

### 3.4 Role of fatty tissue in shaping the TME

Obesity-induced adipose dysfunction increases the risk for breast cancer development and progression by initiating chronic low-grade inflammation because of adipokine secretion. Cancer cells typically invade the adipose tissue and induce adipocytes to release free fatty acids, which are used by cancerous cells to produce ATP and facilitate tumor growth [63]. Adipocytes are the primary cellular component of the breast tumor microenvironment; it contributes to tumor invasion and progression by the secretion of extracellular matrix (overexpression of collagen VI), production of multiple MMPs (MMP-3 and MMP-9) and proinflammatory cytokines. Compared to normal adipocytes, breast cancer-associated adipocytes (CAA), exhibit a series of characteristics, such as fibroblast-like phenotypes, small morphology, dispersed lipid droplets, and low expression of adiponectin [64]. These features of CAA provide drug resistance by modulating apoptosis. Besides chemotherapy, CAA also provide resistance to radiotherapy in breast cancer through increased activation of the effector kinase Chk1 [65]. Adipocytes also inhibit trastuzumab-mediated antibody-dependent cellular cytotoxicity in HER2-expressing breast cancer cells *via* the secretion of soluble factors [66]. Recent evidences show that tumor-surrounded adipocytes provide resistance to *doxorubicin*, a typical chemotherapeutic drug used in breast cancer treatments, as well as to other chemotherapeutic agents [67].

## 4 Breast cancer models

**Fig. 2:**
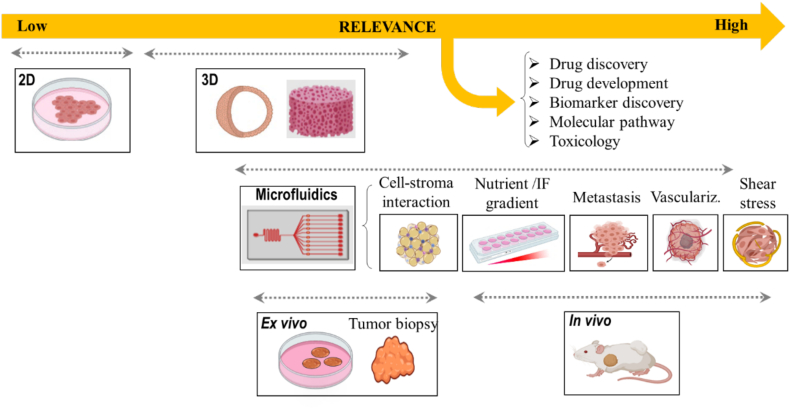
Overview of *in vitro* models for studying breast cancer physiopathology and for drug screening applications. The 2D tumor model is typically represented by a monolayer culture of cells; 3D tumor models (*e.g.*, spheroids, cancer cells encapsulated within scaffolds/hydrogels, microcarriers, and others) can reproduce native cell-cell communication and cell-ECM interactions. *Ex vivo* (tumor biopsy) and *in vivo* models can be used for drug screening, drug discovery and development, biomarkers detection and to indentify molecular pathways involved in breast tumor. Microfluidic chip models can mimic the *in vivo* physiopathology of breast cancer, such as vasculature growth, gradient generation, interstitial flow, or shear stress. In addition, important events of the metastatic cascade can be easily reproduced and studied, such as tumor growth, invasion, intravasation, vasculature CTC transit, extravasation, or organ specificity. (Created using Biorender.com).

A diverse variety of breast cancer models have been reported to investigate the complex physiopathology of the disease. These models are very heterogeneous ranging from standard 2D and 3D cultures to more complex animal models. In the following, we briefly describe the most typical pre-clinical models used for reproducing the complex interplay between the heterogeneous population of cells and the TME during breast tumor progression (Fig. 2). For detailed information, the readers may refer to the specialized reviews on the topics [6,9].

### 4.1 *In vitro* breast cancer models

*In vitro* cancer models include a large plethora of 2D and 3D systems, ranging from simple Petri dishes, tissue culture flasks or transwell plates, to more elaborated scaffolds, hydrogels, or spheroids and others. This type of cell culture techniques is extensively employed to study the complex cellular and molecular mechanisms underlying the breast tumor physiopathology [[68], [69], [70]]. This includes the identification and regulation of novel therapeutic targets and breast cancer-associated markers, such as estrogen [71], progesterone [72], HER2 [73,74], CCL18 [75], miR-31 [76], melatonin [77], or WNT5A [78] and others. Among all the *in vitro* –breast– cancer models, 2D systems are still the preferred platform for the pharmaceutical and biotechnological companies to develop and screen novel therapeutic compounds. This is because 2D *in vitro* tumor models are, in general, easy to use, cheap, and provide highly reproducible results. They can also be mass-produced and are compatible with current technologies, among other advantages. However, they are oversimplified models, and typically lack information regarding cell heterogeneity, extracellular matrix and 3D interactions thus, limiting their applicability and the relevancy of the obtained data.

On the other hand, 3D *in vitro* models widen the spectrum of analysis to cell-cell and cell-ECM interactions [4]. Therefore, key events in the metastatic cascade, such as intravasation, extravasation, tumor-stromal cell invasion, or angiogenesis [81,82], can easily be reproduced using these models. As aforementioned, these types of models include spheroids [83,84], scaffolds [85,86], or hydrogels [87], and others. The more elaborated 3D *in vitro* models also include micro-carriers or decellularized matrices. Most of these models are employed in breast cancer research. There is a transition from using monoculture 3D spheroids to a heterotypic tumor spheroid to study tumor progression or explore tumor therapies. As described earlier, adipocytes are one of the stromal cells, which can interact with breast epithelium, and release variety of cytokines and hormones. However, there is no widespread incorporation of adipocytes in establishing tumor models and the adipocytes used in culture are usually 3 T3-L1 murine embryonic preadipocyte cell line. Therefore, to mimic better the actual scenario, a recent study utilized human mesenchymal stem cells (hMSC) derived from patients to generate adipose tissue to investigate their role on MDA-MB-231 cell migration. The obtained results showed an enhanced migration when using an adipose-containing model over empty scaffolds, highlighting their influence on breast cancer cell migration. Further, it was also suggested that this type of approach may be utilized for personalized therapy strategies by utilizing patient derived tumor biopsies [79].

Finally, improved 3D tumor models based on breast cancer microtissues were independently developed by Brancato *et al* and Mazio *et al*. Both teams used gelatin microporous beads to overcome the major hurdle faced by 3D *in vitro* models of plasticity and heterogeneity. The developed models involve the multiple cell types including MCF-7 cells and fibroblasts. Importantly, this shows the dynamic remodeling of the ECM triggered by the tumor, moreover, reproduced the same events occurring *in vivo* [4,80].

Even though these models reproduce *in vivo*-like features, they still lack important characteristics of the physiological scenario, such as fluid flow, shear stress, mechanical forces, or limited cell heterogeneity [81]. These limitations may threat the relevancy of the obtained data, including the physiological dosing of the tested therapeutic agents or key mechanistic insights [82].

### 4.2 *In vivo* breast cancer models

Animal models overcome most of the limitations of 2D and 3D *in vitro* systems, including the involvement of multiple cell types, fluid flow, mechanical forces, ECM remodeling, or the formation of tumor at secondary metastatic sites. *In vivo* models range from simple model systems, such as *Drosophila, Zebra fish or C. elegans* to more complex models, such as mice, pigs or primates. The most practiced models are murine models, which range from orthotopic or ectopic based on placement of engrafted tumor tissue at the matching or different tissue site. Another class is metastatic cell-derived xenografts, and platelet-derived xenografts, which mostly comprise human tumor xenografts involving the transplantation of human-derived tumor cells into a mouse model. In addition, there are syngeneic, conventional and conditional genetically-engineered mouse models or humanized mouse models based upon genetic information of the mice [83]. Among them, the genetically-engineered mouse models have significantly contributed in breast cancer research by examining crucial squamous cell markers, such as Lgr6 metastatic marker Malat1, or the DNA binding inhibitor ID2 [[84], [85], [86], [87]]. A few studies have shown the progress of utilizing 2D/3D models for drug screening or cellular events investigation (*e.g.,* cell migration or proliferation) to finally recapitulate the effect on *in vivo* models. In this regard, triple negative breast cancer cell line has been employed to study the effect of 20 phytochemicals on cell migration and select the best compound to be tested in a 3D tumor spheroid model [88,89]. It was found a reduced matrix invasion in the latter case, further establishing the role of the selected drug, *fisetin*, in reducing tumor metastasis in a zebrafish tumor model.

This type of models is very time consuming in the pipeline of drug discovery and screening. In addition, the selected drug candidates still need to be clinically validated in humans. Therefore, despite their advantages, the absence of human cells does not result in direct clinical translation. On the other hand, patient-derived xenografts (PDXs) have gained much attention as they recapitulate the genomic and transcriptomic information of the original tumor. Therefore, they can serve as an important tool in therapeutic testing [90]. As an example, Cottu *et al* identified the role of PI3K in acquired hormone resistance associated with PDX models of luminal breast cancer [91]. Another study showed the increased breast cancer stem cell activity through JAG1-NOTCH4 receptor activation as a possibility towards acquired resistance to hormonal therapies [92]. The importance of anti-immune response for tumor regression was also evaluated in an orthotopic (4 T1 cell-induced) mice model after vaccination with drug-treated tumor cells [93]. The inter-model differences in miRNome profile between 4 T1 injected- orthotopic and intravenous (IV) models indicated the potential of finding candidate genes responsible for metastasis in different models [94].

Despite the intrinsic advantages exhibited by *in vivo* models, they suffer from certain limitations, including high cost, time-consuming, ethical concerns, reduced reproducibility, or limited manipulation. Importantly, these models are not predictive about the outcome of therapeutic drugs in humans. Also, the invasion of stromal cells of mouse over time and immune cell interaction still raises concern in PDX [95].

### 4.3 *Ex vivo* breast cancer models

Biopsy samples allow the study of a heterogeneous population of cells, which maintain the cell-cell interactions and microenvironmental cues of the native tissue. *Ex vivo* samples can be utilized to identify or validate a diagnostic or therapeutic marker. As an example, Andrade *et al* studied the influence of platelet rich plasma as one of the TME components to mimic better the *in vivo* scenario. Stromal and tumor cell population from 21 women with different breast cancer subtypes was isolated. The plasma components showed a tumor subtype (luminal A and B, and HER2+ breast cancer subtype) and cancer cell type (epithelial and stromal cell) specific influence upon tumor progression and cytokine profile [96]. Likewise, a study utilized breast tumor tissue samples to identify better processing methods to perform single cell RNA sequencing. They found minimum alterations associated with cold temperature while other methods mostly resulted in activation of certain stress response [97]. Another work reported an NGS panel (MammaSeq™) to identify clinically actionable mutations in solid tumor and circulating tumor DNA (ctDNA) isolated from 46 and 14 patients, respectively. The panel identification percentages were 48% and 29% in solid tumor and ctDNA, respectively [98]. Patient-derived samples also help to establish the role of bioinformatically-derived potential targets. For example, CXCR4/CXCL12 signaling pathway was identified by The Cancer Genome Atlas Program (TCGA) analysis. Its immunosuppressive role was confirmed by using CXCR null cell line and a murine metastatic breast cancer model [99]. However, the biopsy sample does not represent the entire tumor components, and in particular, the rheological properties of the TME.

## 5 Tumor-on-chip models

During the last decade, the combination of tissue engineering approaches, nanotechnology tools, and cell biology concepts, resulted in the development of a new generation of physiologically relevant *in vitro* models. They reproduce the functional units of a human organ or tissue inside a microfluidic chip. These models are denoted as “organ-on-chip” and reproduce all the cellular, biological, and structural features of the native scenario. Importantly, they also mimic the main dynamic events occurring *in vivo*, such as fluid flow, shear stress, nutrients supply, or waste removal, among others. The integration of cancer cells into this type of microfluidic devices results into cancer- or tumor-on-chip models. They recapitulate better tumor pathogenesis, and therefore provide a physiologically-relevant environment for mechanistic and drug discovery/screening applications [100]. Tumor-on-chip systems can also control the internal and external stimuli such as, dynamic mechanical stress, interstitial fluid pressure, or concentration gradients. They can also recapitulate physiological flows and cell heterogeneity to simulate the biomechanical and cellular complexity of the native tumor. As a result, this facilitates the evaluation of the efficiency of anti-cancer drugs [101]. In the following, we highlight the main advantages of organ-on-chip models in breast cancer research compared to conventional *in vitro* (2D and 3D) and *in vivo* systems (see also Table 1).

**Table 1:** Comparison between conventional *in vitro* (2D and 3D), *ex vivo*, *in vivo* models and microfluidics systems.

Characteristics	2D culture	3D culture	*in vivo*	*ex vivo*	Microfluidics
*Ease of assay*	Easy to perform	Difficult to form uniform 3D models	Requires specialization. It is also laborious and time-consuming	Requires optimization. It is time consuming	Requires specialized equipment for chip fabrication and trained personnel
*Time required*	Low	Moderate	Very high	Very high	Moderate
*Reproducibility*	High	Moderate	Low	Low	High
*Cost*	Low	Moderate	Very expensive	Expensiv	Moderate (The assays are cheap but expensive equipment is needed)
*High throughput screening*	Possible	Possible	Not possible	Not easy	Possible
*Main applications*	Invasion, proliferation, cell-signaling, drug response studies	Invasion, cell-cell/matrix interactions, intra and extravasation, hypoxia, drug response	Metastasis, drug response, mutation studies	Anticancerous drug testing and biomarkers discovery	Multicellular interactions and recapitulation of *in vivo* conditions such as vasculature, fluid flow, biochemical gradient is possible; can incorporate immune cells; provides an ethically relevant substitution of *in vivo* model
*Sample volume requirement*	Low	Low	High	High	Very low
*Biological relevance*	Limited relevance (cell display artificial phenotypes and perturbed gene expressions)	Higher biological relevance (compared to 2D)	Very high biological relevance (compared to 2-D and 3-D); Provides physiological microenvironment and vasculature;	Higher biological relevance	Very high biological relevance
*Main limitations*	Lack of vasculature and cell-matrix interactions Lack of perfusion	Lack of vascularization. Lack of perfusion	Mostly suffer to demonstrate immunomodulatory effect. Non-predictive	Lack of vasculature and perfusion. Short observation period.	Difficult to collect cells for analysis

### 5.1 Microdevices to mimic the tumor microenvironment

Breast cancer progression is modulated by a complex interplay of cellular, genetic and epigenetic factors. In the primary tumor, cancer cells grow uncontrollably and interact with the neighboring stroma and ECM components. In the TME, hypoxia, growth factor gradients, aberrant vasculature, and the interaction of cells with the stromal components are some of the main aspects, which contribute to the dissemination of the tumor to distant tissues. Tumor -on-chip systems are well suited to model and monitor these and other key events, improving our knowledge in breast cancer biology and therapeutic responses.

#### 5.1.1 Gradient generation

**Fig. 3:**
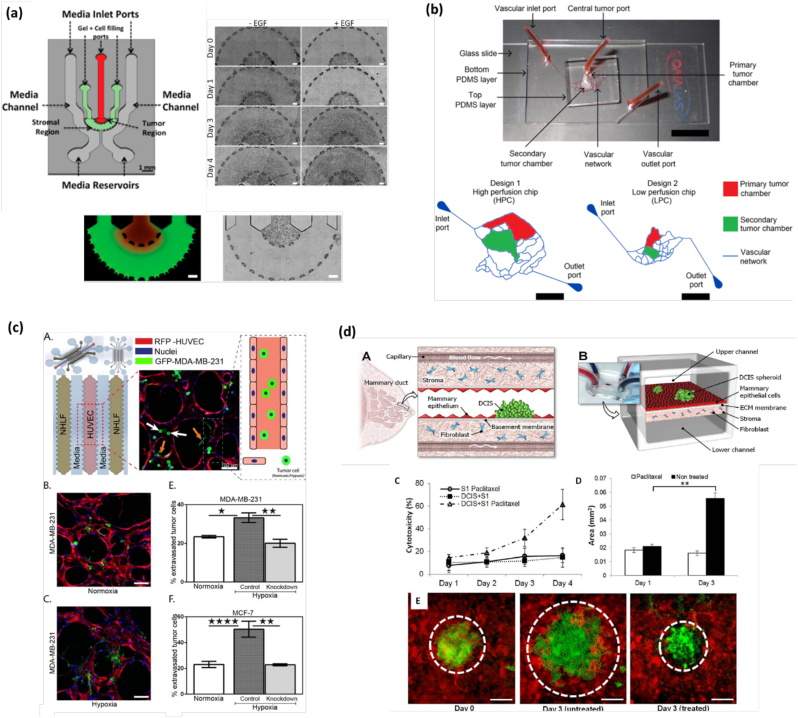
Human breast tumor-on-a-chip models. (a) Mimicking gradient generation on-chip. *(Left)* Microfluidic device for the study of breast cancer cell invasion into the 3D stroma. Bottom images show the spatial organization of cells encapsulated within a 3D matrix. (Right) Time-sequence for 4 days showing the invasion of EFG+ and EGF- SUM-159 breast cancer cells into the neighboring stroma. Reproduced with permission from [5] (Creative Commons Attribution 4.0 International License). (b) Mimicking fluid dynamics on chip. *(Top)* Tumor-mimetic microfluidic chip containing a realistic vascular network. *(Bottom)* Schematic representation of the vascular network, primary and secondary tumor chambers. Reproduced with permission from [105] (Creative Commons Attribution 4.0 International License). (c) Mimicking hypoxia effect on-chip. (A) Microfluidic chip showing the distribution of NHLF, HUVEC, and invasive GFP-MDA-MB-231 breast cancer cells. (B—C) Immunofluorescence image under normoxia (B) and hypoxia (C) conditions. (*E*-F) Quantification of the % of extravasated tumor MDA-MB-231 (E) and MCF7 (F) cells for all conditions. Reproduced with permission from [107] (Creative Commons Attribution 4.0 International License). (d) Mimicking tumor-stroma interactions on-chip. (A) The Ductal caricinoma *in situ* (DCIS) is embedded in a mammary duct consisting of the mammary epithelium and a basement membrane surrounded by stromal tissue (fibroblasts). (B) The microarchitecture of the DCIS and the surrounding tissue layers is reproduced in the breast cancer-on-a-chip microdevice comprised of the upper and lower cell culture chambers separated by an ECM-derived porous membrane. (C) cells are treated with *paclitaxel* from the basal side to simulate intravenous administration. (D) *Paclitaxel* treatment prevents growth of DCIS spheroids (white). (E) Fluorescence micrographs of DCIS spheroids at day 0 (left), day 3 without *paclitaxel* (middle), and 3 days with *paclitaxel* treatment (right). Reproduced with permission from [16] (Creative Commons Attribution 4.0 International License; CC BY 4.0).

Cell migration and invasion are the first events in the cascade of tumor progression. These phenomena are driven by various gradients of growth factors, chemoattractant and other biological and mechanical cues. Microfluidic devices can easily reproduce this type of biochemical gradients by two main different methods: *flow*- and *diffusion*-based [5]. The flow-based gradient is mostly dependent on the presence of fluid flow over the gradient regions, utilizing the convection in the laminar flow streams to form a molecule gradient. In contrast, the diffusion-flow method mainly depends on the diffusion of soluble molecules through the microchannels with high fluidic resistance or within 3D matrices [102]. Designing special microfluidic chips having one central and two side channels interconnected by small pillars can create a gradient flow. One of the lateral channels is used to inject the drug (or other signaling molecules) while the other is typically filled with culture media. These microchannels allow the diffusion of compounds through the pillars but do not allow a substantial fluid flow from the lateral channels to the central one [103]. In this line, Truong *et al*. monitored the effect of EGF on the invasion of SUM-159 breast cancer cells [5] (Fig. 3a). The culture medium containing EGF (50 ng/ml) was added to the microfluidic chip for 24 h. The EGF stimulated and non-stimulated SUM-159 cells were tracked for a period of 4 days. During the first 24 h, the cells remained inside the tumor region, whereas after 24 h, EGF stimulated cells started invading the stromal region. The breast cancer cells, after 4 days stimulated by EGF+ invaded tumor farther than EGF- [5]. Similarly, Islam and Resat used a microfluidic device to culture MDA-MB-231 breast cancer cells finding that their motility depended on the concentration and gradient of EGF [104]. They divided the cells into four groups as per their exposure to EGF gradient ranges, low (0–9.5 ng/ml/mm), medium (9.5–19 ng/ml/mm), high (19–28.5 ng/ml/mm), and very high (28.5–38 ng/ml/mm). The obtained results showed a clear increase in the velocity of cell migration upon ligand gradient exposure. It was concluded that the ligand concentration by itself did not show much impact, but the ligand gradient was the main factor to enhance breast cancer motility.

#### 5.1.2 Mimicking the fluid dynamics of the tumor microenvironment

Microfluidic systems are capable to mimic the *in vivo* fluid dynamics of the TME by providing a continuous perfusion of nutrients and oxygen as well as by the removal of waste product. This ensures proper cell viability and system homeostasis. As an example, Lang and colleagues seeded MDA-MB-453 breast cancer cells in three different ECM matrices (Matrigel™, BME2rgf, and collagen I) under static and perfused conditions [21]. In the perfused system, cells showed a higher viability than in static culture. Similarly, cell growth rate remained at 80% in the perfused system compared to static culture (60%). Pradhan *et al*., fabricated a high and low perfusion chip based on comparative degree of fluidic exchange between the lateral microvascular and central tumor channels to mimic cancer-ECM-endothelial interactions (Fig. 3b) [105]. The high perfusion chip provided a higher shear rates (40–50 s^−1^) compared to the low perfusion chip (10–20 s^−1^). In the high perfusion area, MCF7 and MDA-MB-231 cells elongated forming colonies similar to *in vivo* condition, while in the low perfusion region, both cell types were rounded and dormant due to insufficient nutrient availability [105]. Similarly, fibroblasts located in the high perfusion regions displayed elongated phenotypes, whereas in the low perfused region they were rounded and dormant. These cellular features illustrate the importance of perfusion to mimic the native condition, where nutrient rich zone contains live and proliferative cells while nutrient deficient regions exhibit necrotic cell death [105].

#### 5.1.3 Mimicking the biochemical and metabolic properties

Hypoxia is associated with tumor growth and metastasis and contributes to drug resistance. To explore the role of hypoxia in tumor progression and drug resistance, a breast-on-chip model was recently developed containing 3D breast tumor spheroids [106]. The cells were exposed to controlled spatiotemporal oxygen concentration to mimic the hypoxia condition in solid tumor. The effect of precise oxygen control towards cell behavior provided the information about swelling and shrinking nature of tumor spheroids. The tumor spheroids also showed heterogeneity in *doxorubicin* uptake [106]. It was reported that this platform could be used for the screening of cancer treatments under controllable hypoxic conditions. Next, Song et al., developed a microfluidic breast-on-chip platform containing three-gel channels to investigate effect of hypoxia on cancer progression (Fig. 3c) [107]. HUVECs were seeded in middle channel and normal human lung fibroblasts (NHLF) were seeded in the lateral ones to stabilize the vasculature. Breast cancer cells were cultured in normoxic or hypoxic condition for 5 days and were afterward introduced into the microvascular networks on day 4, to mimic the TME [107]. This investigation showed that under hypoxia condition HIF-1α level is elevated. This changed the cell morphology, viability, extravasation rate and metastatic potential, which further assisted in cancer progression. In a different study, breast cancer cells (MCF7 or MDA-MB-231) and immune cells (THP-1) were encapsulated within a 3D hydrogel with endothelial cells (HUVEC) seeded in the lateral channel of a microfluidic chip to create varying levels of hypoxia. Upon hypoxia stimulation, the tumor cells displayed higher levels of chemokines CCL5 and CCL20, which promoted cancer progression and metastasis [108].

Organ-on-chip models are also employed to study the metabolic properties of cancer cells, such as glucose consumption and lactate production. In this regard, a droplet-based microfluidic device in combination with 18F-fluorodeoxyglucose-radioluminiscence microscopy was used to characterize the metabolic profile of single breast cancer cells by utilizing 18F-fluorodeoxyglucose consumption and lactate production. The quantitative measurement was carried out by monitoring the uptake of radiolabeled molecules by single cell droplets encapsulation. This technology can be used as alternative to Warburg for metabolism-based cancer screening [109]. Another study was carried out on DCIS model to monitor the biochemical and metabolic properties of breast cancer. The DCIS model was created by using normal mammary cells to generate the mammary duct; two flanking lumens were used to perfuse media, metabolites or drugs. This model exhibited hypoxia generation, rapid consumption of glucose, glutamine and lactose secretion. It also manifested a higher expression of hypoxia related CA9 gene to regulate the intracellular pH, which promoted cell survival under toxic pH conditions [110]. These biochemical metabolic analyses of tumor cells can be used to unveil the mechanisms behind tumor heterogeneity and energy metabolism. They are significant to provide the clues about the metastatic potential of the tumor and its resistance to drug treatment [6].

#### 5.1.4 Modeling the vasculature: intravasation and extravasation

Organ-on-chip devices can mimic the complex *in vivo* hemodynamics of the TME by reproducing the native microvasculature. This allows to study the mechanistic determinants and the effect of therapeutic drugs during the intravasation and extravasation of cancer cells [111]. These are two fundamental events in the metastatic cascade after the invasion of the surrounding tissue by tumor cells. Next, these cells intravasate the vasculature (blood and lymphatic vessels) where they transit along the vasculature as circulating tumor cells (CTCs). Eventually, these CTCs arrest in the vessel walls where they transmigrate the endothelium to extravasate. Finally, cancer cells invade the metastatic target organ, wherein tumor cells establish and develop as micro and macro metastasis (Fig. 1). A clear understanding of the mechanism at work of all these interconnected processes may provide novel potential therapeutic approaches. In this regard, several organ-on-a-chip models are developed to study all the events in the cascade of metastasis. Among all of them, the intravasation and extravasation of cancer cells can be considered as the most important events. Herein, we briefly discuss about some of the most relevant intra- and extravasation-on-a-chip models in breast cancer.

##### 5.1.4.1 Tumor intravasation-on-a-chip

Intravasation is a crucial process for the progression of distant metastasis. During intravasation, cancer cells invade the blood or lymphatic vessels near the tumor stroma triggered by chemotactic gradients (*e.g.,* growth factors), oxygen tension, and diminished endothelial barrier [112]. To gain insights about the mechanism of intravasation, different breast tumor-on-chip models have been reported. As an example, Lee *et al*.*,* described a miniaturized microfluidic model to explore the relationship between breast tumor-stromal and breast tumor-endothelial interactions. The model was employed to examine the effect of *bevacizumab*, an antibody targeting the vascular endothelial growth factor protein, on tumor angiogenesis, finding a drastic reduction in the number and coverage area of vessel sprouting. They also investigated the effect of tumor necrosis factor-α on tumor modulation and intravasation [113]. They found that *bevacizumab* treatment drastically reduced the number and coverage area of the micro vessel sprouts. The obtained results revealed that this model might be appropriate for the evaluation of therapeutic compounds targeting cancer angiogenesis. Similarly, Nagaraju and co-workers developed a 3D microfluidic platform with MDA-MB-231 breast cancer cells to investigate their intravasation in well-controlled conditions [114]. In this work, VEGF was added to the culture medium to assess the subsequent effects on vasculogenesis. The results indicated that in the presence of highly metastatic cancer cells, the vascular network was thinner and highly permeable. This demonstrated that with increased VEGF production, vascular leakage assisted more trans-endothelial migration of cancer cells. Likewise, Wong and Searson developed a perfusable artificial vessel comprised of endothelial cells and single and clusters of MDA-MB-231 breast cancer cells in a 3D collagen matrix [115]. Live-cell fluorescence microscopy was used to monitor the invasion, intravasation and tumor-ECM-endothelial interactions during the cancer progression recapitulating many features of the distinct tumor niche within a microenvironment. These findings might be helpful to understand tumor cell interactions with the vascular network and to unveil the biological mechanism involved during invasion and intravasation. Finally, these discoveries provide new insights about the mechanism of tumor metastasis and come up with a way to explore the efficacy of anti-tumor drugs in a physiologically relevant environment.

##### 5.1.4.2 Tumor extravasation-on-a-chip

Tumor extravasation refers to the transmigration of CTCs through the endothelial barrier of the vasculature and lodging at the secondary organs [111]. As an example, a breast tumor-on-chip model was reported to analyze the mechanism of extravasation of MDA-MB-231 human breast cancer cells into bone- and muscle-mimicking microenvironments through a microvascular network concentrically wrapped by mural cells [116]. The extravasation rate of breast cancer cells was significantly higher in the bone-mimicking microenvironment compared to the control and to the muscle-mimicking counterpart. The addition of the pro-inflammatory cytokine TNF-α enhanced the microvasculature permeability and cancer cell extravasation in a dose-dependent manner [117]. In this line, many researchers indirectly targeted signaling molecules, which induced the extravasation process. As an example, Chen *et al*., developed a breast tumor-on-chip platform to investigate the role of integrin β1 in the extravasation potential of breast cancer cells [118]. Small hairpin RNA targeting integrin β1 caused a significant reduction in MDA-MB-231 cell invasive protrusions and extravasation in 6 h. They also reported that co-blocking of laminin-binding integrin α3 and α6 reduced tumor extravasation. In another study, a microfluidic device was utilized to create a 3D microvascular model of breast cancer seeded under different oxygen condition to explore the role of HIF-1α in tumor extravasation. However, after siRNA knockdown, the expression of HIF-1α significantly decreased reducing the rate of extravasation, which may have an impact on apoptotic and metastatic-related cellular process [107].

It is known that bone and brain are the preferred sites where breast cancer cells metastasize after extravasation. A microfluidic device containing one channel to grow bone cells and another one for endothelial cells (HUVEC) was reported to investigate the metastasis from the breast to the bone [116]. The bone channel consisted of osteo-differentiated human bone marrow-derived mesenchymal stem cells (hBM-MSCs) seeded within the collagen gel. Osteogenic medium was supplied for 3 days to induce bone formation. Next, HUVECs were cultured in another channel coated with Matrigel™. After 3 days, MDA-MB-231 breast cancer cells were seeded with HUVECs. A significant increase in their extravasation rate and migration distance was observed compared to simple collagen gel without hBM-MSCs. Likewise, the metastasis of breast cancer cells to the brain was investigated using a microfluidic model. The complex blood brain barrier (BBB) microenvironment within the chip was created by using astrocytes and human bone marrow microvascular endothelial cells *via* physical cell-cell interaction, vascular mechanical cues and cell migration. To observe extravasations of breast cancer cells, MDA-MB-231 cells were injected into the middle channel, which eventually adhered the BBB [119]. Overall, these two studies illustrate how microfluidics devices are capable to reproduce the main events occurring during extravasation and trans-endothelial migration of cancerous cells and its inhibition [120].

#### 5.1.5 Modeling tumor-stroma interactions

Adipocytes are the primary cellular component of the breast tumor microenvironment. It contributes to tumor invasion and progression by the secretion of MMP3 and pro-inflammatory cytokines [63]. Breast cancer-associated adipocytes provide resistance to drugs, chemotherapy and radiotherapy [64]. Thus, it is very important to understand the underlying mechanism by which adipocytes contribute to treatment resistance. In this line, Yang *et al*., mimicked the *in vivo* heterogeneous cancer microenvironment by using a 3D breast tumor-on-chip device [121]. MCF7 breast cancer cells were co-cultured with primary adipocytes finding that the formers were more resistant to photodynamic therapy than in 2D conditions. Additionally, CAAs were also found to stimulate breast cancer cell migration, invasion and drug resistance. Similarly, Crake *et al*., isolated adipocytes from human breast adipose tissue and co-cultured them with hormone receptor-positive MCF7 and triple-negative MDA-MB-231 breast cancer cells. They observed predominant down- and up-regulation of highly differentially regulated proteins. This supports the concept of reciprocal communications between breast cancer cells and CAAs. Overall, this investigation provided a better understanding of the molecular mechanisms by which cancer-associated adipocytes regulate breast cancer cell phenotype and function. It also provided a platform for the identification of novel protein targets involved in breast cancer migration and metastasis [122].

Solid evidence has shown the important role of CAFs in tumor progression. Recently, a 3D microfluidic device was developed integrating breast cancer cells to unveil the molecular influence of tumor-stromal interactions on metastasis [123]. The obtained results showed that CAFs enhanced breast cancer cell migration and invasion speed by inducing the expression of the novel gene glycoprotein non-metastatic B. This model provided important insights about the cellular and molecular consequences of tumor-stromal interactions in tumor microenvironment. Similarly, Gioiella *et al*., reported a breast tumor-on-chip device where normal and stromal epithelial cells were separated by an interface to simulate the cancerous epithelial-stromal interaction. In this study, normal and CAFs were used to produce cancer microtissues. When these cells were co-cultured, the normal fibroblasts were differentiated into myofibroblasts after their interaction with the cancer cells [124]. This work also evaluated the expression of MMPs during cancer invasion. The paracrine signaling between the cancer cells and fibroblasts induced the production of MMP2 and MMP9, which degraded the collagen IV and weakened the basement membrane. The degradation of the ECM provided the needed signaling cues to regulate tumor cell migration.

This type of co-culture models holds very promising for drug screening applications since cancer cells respond to therapy in a similar way they do *in vivo*. In this regard, Choi *et al*.*,* designed a micro-engineered 3D pathophysiological model of breast ductal carcinoma *in situ* (DCIS) by culturing breast tumor cells with mammary fibroblasts and mammary ductal epithelial cells. The model was employed to evaluate the effect of typical anti-cancerous drug, *paclitaxel* (Fig. 3d) [16]. After drug exposure, the diameter of the DCIS spheroids remained unchanged or slightly decreased. This finding shows that the pathophysiological model may be useful for better understanding of DCIS progression and for the development of new therapeutic treatments. Tumor-associated stromal components are not only significantly to elevate the treatment efficacy but also increase the treatment depth and uniformity.

## 6 Applications of breast tumor-on-chip technology

Breast tumor-on-chip systems can be applied for a multitude of applications, ranging from mechanistic studies to drug screening or discovery (Table 2). This technology provides the opportunity for the rapid diagnosis of the disease and screening of anti-cancerous drugs, for disease modeling, or for the detection of new biomarkers and therapeutic approaches. In addition, the integration of multiple tissues and high throughput multi-data analysis into microfluidics further improves the disease detection and diagnosis sensitivity and accuracy. In the following, we describe the main applications of breast tumor-on-chip systems in disease detection, diagnosis, modeling and high throughput data analysis.

**Table 2:** Summary of breast tumor-on-a-chip models.

Applications/objectives	Cancer cells used	Flow type	Media exchange method	Ref.
*2D culture of cells*				
Staurosporine related chemo-sensitivity of breast cancer cells	MCF-7 (ER+)	Normal	No media exchange (separate chips for different time points)	[154]
Local vascular dynamic modeling	MDA-MB-231	Normal	Perfusion	[155]

*2D + co-culture of cells*				
Changes in gene expression level, while transitioning from 2D to co-culture	MCF-7	Normal	Direct exchange of media	[156]

*3D culture of cells*				
*Ex-vivo* drug screening with mimicked vascular flow	T47D	Gradient	Perfusion	[157]
Model for understanding extravasation of circulating tumor cells	MDA-MB-231, MCF-7	Gradient	Perfusion	[158]
Live cell imaging platform for intravasation	MDA-MB-231	Gradient	Perfusion	[117]
Micromolded hydrogel-based 3D culture	MCF-7	Normal	Direct exchange	[159]
TME model for studying EPR (enhanced permeability and retention) effect of rapid drug screening	MCF-7, MDA-MB-231	Gradient	Perfusion	[160]
Microfluidic model for simulating differential response of *doxorubicin*	MDA-MB-231, MCF-7	Normal	Perfusion-based media delivery	[139]
Models for understanding breast cancer metastasis	MDA-MB-231	Gradient	Direct	[115]
Role of interstitial fluid pressure in regulating invasion in engineered breast tumors	MDA-MB-231	Gradient	Direct exchange; Perfusion	[161]

*3D + co-culture of cells*				
Stratified 3D culture of cells	4 T1, 3 T3, HepG2	Normal	Direct media exchange	[162]
Pathophysiological model for early stage breast cancer	HMF, DCIS	Gradient	Perfusion	[18]
Microfluidics-based simultaneous culture of multiple cell lines	MDA-MB-231	Normal	Direct exchange	[163]
Tumor cell interactions with microvasculature for trans endothelial migration study	MDA-MB-231, HUVEC	Gradient	Perfusion	[117]

### 6.1 Detection of breast cancer biomarkers

**Fig. 4:**
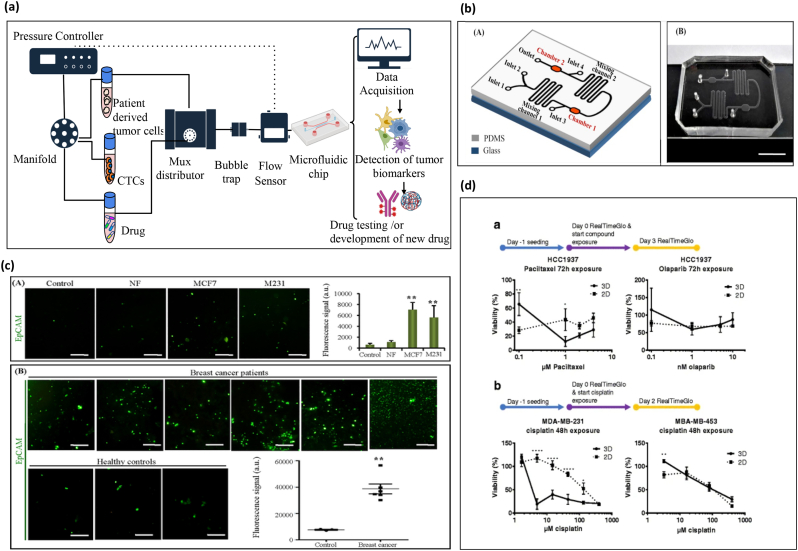
Overview of tumor therapy approach through microfluidic technology for drug screening and breast tumor marker detection and quantification. (a) Schematic representation of microfluidic setup. used for the sorting of circulatory tumor cells and breast tumor specific biomarkers from solid tumor and patient-derived samples . A A pressure controller and a flow sensor (MFS) are used to create a precise interstitial fluidic pressure and flow speed, similar to the perfused native cancerous tissue. It can be used for biomarker detection and drug treatment. (b) Schematic representation and image of the microfluidic chip for exosomes capture and detection (A and B). (c) (A) Quantification of EpCAM-positive exosomes from breast cancer cell lines from control, normal fibroblast, MCF7 and MDA-MB-231 culture medium. (B) Quantification of captured EpCAM-positive exosomes from plasma samples of breast cancer patients and healthy controls, Reproduced with permission from [146] (Creative Commons Attribution 4.0 International License). (d) Screening studies of breast cancer cell lines in 2D and 3D microfluidic culture (*bottom left*). a, HCC-1937 (TNBCs) were seeded in 3D OrganoPlate® and 2D tissue culture plates. *Paclitaxel* and *olaparib* drugs were added after 72 h. b, Similarly, MDA-MB-231 and MDA-MB-453 cells were seeded and exposed to *cisplatin* at various concentrations and cellular viability was quantified. Reproduced with permission from [21] (Creative Commons Attribution 4.0 International License).

The gold standard for the detection and diagnosis of breast cancer includes the use of mammography, sonography, computerized tomography, biopsy, and magnetic resonance imaging [125]. These diagnostic procedures have certain limitations, such as time consuming, expensive, and not appropriate for young women. These have denser and less fatty breast compared to older women. Mammograph of young women with dense tissue may have a dominant whitish appearance and the appearance of cancerous or abnormal cells are also white. Consequently, it is challenging to interpret the result. Microfluidics can significantly contribute to the early and reliable diagnosis of breast cancer by the detection of predictive biomarkers, such as circulating tumor cells, DNA/RNA or antibodies, in a process denoted as liquid biopsy (Fig. 4a).

There are well-established diagnostic biomarkers (ER, PR and HER2) of breast cancer currently used in the clinics. Estrogen receptor-alpha (ER-α) is one of the potential targets in breast cancer treatment [152]. It is well known that estrogen plays an important role as autocrine and paracrine messenger in most of the tissues, including breast. The activity of ER-α is activated upon binding to its ligand, estrogen, which is the primary therapeutic target in breast cancer. Therefore, it is important to evaluate the estrogen concentration in breast tissue to identify tumor development or to monitor the anti-estrogen treatment strategy [126]. Few soluble factors are implicated in ER-α activation (progesterone receptors; PR, pS2, TFF1), and suppression (ER-α, ESR-1). There are two major classes of hormone therapy used in the treatment of ER-α positive breast cancer. They target the estrogen-dependent but not the estrogen independent activity. It is widely considered that the estrogen independent activity of ER-α underlies therapy resistance. In this direction, a conventional cell culture model was developed to monitor the effect of ER-α treatment on MCF-7 breast cancer cells. This cell line closely recapitulates estrogen-dependent growth and ER-α activation and regulation seen *in vivo*. The large media volume diluted the secreted biomarkers, which create the question of reproducibility and accuracy of this system. In contrast, miniaturized microfluidic chambers are able to culture breast cancer and stromal cells (MCF-7 and HS-5) with microliter volume and they are able to control paracrine signaling of ER-α with great accuracy and sensitivity. Measurement of this dynamic biomarker in miniaturize system may help in hormone therapy response [127].

Metastasis and tumor growth are basically linked to blood circulation, which is used to transport cells (CTCs), cell-free ctDNA, RNA and tumor-derived exosomes. The CTCs are rare and if they are alive in the blood, they may cause metastasis. These CTCs can be used as alternative to invasive tissue biopsy, which is expensive and painful. Therefore, liquid biopsies of blood have great probability to identify and evaluate breast cancer biomarkers for early disease detection, monitoring and diagnosis.

Several microfluidics-based approaches are utilized for the isolation of CTCs and its derived products like membrane trafficking proteins (annexin, Rab GTPases), ctDNA and RNAs (mRNA, miRNA, lncRNA) from liquid biopsy for detection and diagnosis of breast cancer. These devices can also be utilized to monitor the pharmacokinetic and pharmacodynamic responses to understand the drug responses to human body. Currently, FDA approved circulating tumor cell kit (CELLSEARCH®) to monitor the progression of breast, colon and prostate tumors. Several companies, such as Celsee, Biofluidica, Rarecells are developing sensitive microfluidic devices to isolate and characterize the CTCs heterogeneity. The microfluidics devices integrated with immunomagnetic strategy to capture the CTCs from liquid biopsy based on antibody targeting strategy for cell surface associated signaling factors (EpCAM, Trop2, Her2 and Muc1) [128,153]. In this line, Jessen Diagnostic develops the commercialized CTC-iChip based on immunomagnetic-based technology to isolate CTCs from clinical samples (lung, prostate, pancreas, and breast). This CTC-iChip used anti-EpCAM and anti-CD45 coated microbeads (1 μm) based positive and negative methods to isolate CTCs. EpCAM-based positive isolation methods were based on immunoaffinity, which shows high selectivity and specificity for CTCs from blood [129]. Negative methods were not dependent on size and surface-marker, it relies on cellular and transcriptomic biomarkers of cancer. Likewise, positive (EpCAM) and negative (Anti-CD45/CD66b) method used by another group to isolate CTCs from blood [130]. The positive isolation method indicates higher number of CTCs retrieval rates, which increase the EpCAM expression level. In contrast, the negative method shows higher recovery rate (83.1%). These studies display that negative CTCs isolation method was better than the positive, which could be used for the discovery of cellular and transcriptomic biomarkers for cancer [130]. Epithelial derived cell adhesion molecule (EpCAM) biomarkers (CA 15–3, CA 27.29) from breast cancer are also detected by a microfluidic system from blood by using immunomagnetic separation method. The obtained results showed a 90% of sensitivity with a > 95% accuracy [131].

Breast cancer patient's manifest overexpression of miRNA (miRNA-155, miRNA-23, Onco- miRNA) in blood serum. In some cases, when the tissue specimens are not available, these markers may provide useful information about the breast cancer phenotype at an early stage. To evaluate the capability of organ-on-chip models to detect this biomarker, Salim *et al* reported a microfluidic platform attached with a fluorescence reader to explore the role of miRNA in breast cancer. Interestingly, the obtained results were similar to those procured with quantitative real time polymerase chain reaction, suggesting that the developed device could be utilized as a point-of-care diagnosis tool for the early detection of breast cancer stage.

Perturbation in the levels of expression of specific proteins is also employed for the detection of breast cancer. This approach is recently explored using a microfluidic immuno-array for the rapid and low-cost detection of a carbohydrate (CA153 and CAA155) and carcinoembryonic antigen (CEA 153) in breast cancer. For this, an array of primary antibody specific to this protein was bonded to an antigen, which was immobilized on a solid substrate. Next, magnetic particles were conjugated with polyclonal antibodies and peroxidase enzymes and used for breast cancer biomarker detection. The result procured was similar to that of using a commercial electro chemiluminescence kit [118].

Tumor derived exosomes from saliva, breast milk, serum, and plasma are known to involve in breast cancer metastasis. The molecular signature of tumor cells is enriched in exosomes and tumor cells may release more exosomes into microenvironment than normal cells [132]. Microfluidic integrated with optical trappings, electrophoresis, dielectrophoretic and immunocapture technology has been reported for the detection of cancer specific exosomes (CD9, CD63, CD53, CD23, EpCAM and, HER2-positive) from blood plasma of breast cancer patients [133,134]. The exosomes detected by these methods show great efficacy and accuracy. The expression of cancer specific exosomes in patient blood plasma were almost consistent to the solid tumor tissue [135].

Overall, the above-mentioned works exhibit that the microfluidics and organ-on-chip technology can be employed for the accurate, rapid and inexpensive detection of breast cancer biomarkers.

### 6.2 Drug screening

**Fig. 5:**
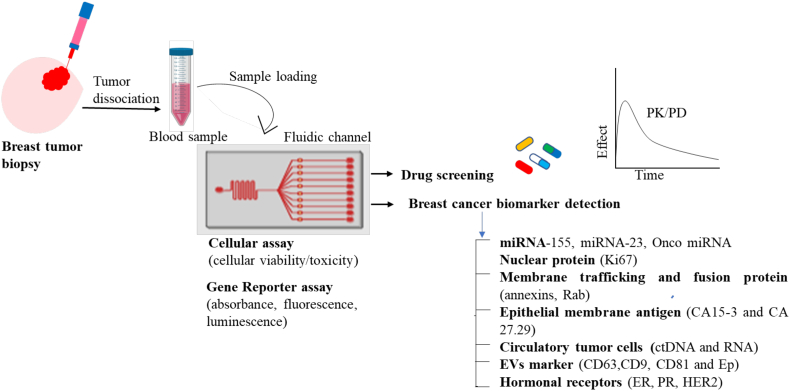
Workflow for a drug screening setup with breast tumor biopsy sample. Blood samples are transferred to the microfluidic chip and incubated with chemical compound libraries. The most efficient drug combination should be determined based on different breast cancer biomarker and phenotypic data. The effect of compounds on blood samples is measured using a variety of cell-based readout and pharmacokinetics/pharmacodynamics (PK/PD) assays. These detection assays can be used for monitoring the cell viability/toxicity and cellular function by the measurement of homogenous changes in absorbance, fluorescence- or luminescence-based gene reporter assays. PK/PD can provide information to simulate drug responses in the human body.

Reproducing *in vitro* the characteristics of the native breast TME with tumor-on-chip models may open new avenues in the field of anti-cancer drug screening (Fig. 5) [116,136,137]. This is of particular interest for pharmaceutical companies who aim at improving the efficiency of drug discovery and screening pipelines at economical way. Typically, a high-throughput droplet-based microfluidics is employed for screening the effect of drugs on individual cancer cells due to the parallelization of experiments related to drug efficacy that can simultaneously be performed [138,139]. Alternatively, 3D tumor spheroids encapsulated within a 3D hydrogel matrix can also be employed to mimic on-chip the properties of the native tumor [16,140].

Drug resistance is a critical phenomenon, which threatens the prognosis of cancer patients. However, the mechanism involved in the development of drug resistance is not yet well understood. Based on the recent developments, the tumor-on-chip models can provide important insights to devise better drug therapies. As an example, the effect of *doxorubicin* (DOX), a typical chemotherapeutic used in breast cancer was compared in a breast tumor-on-chip and a standard 2D model. A higher drug resistance and cell type dependence of DOX was observed in the microfluidic chip compared to 2D culture. This suggests the biasness of an incomplete information provided by 2D studies [141]. Another study used triple-negative breast cancer cells, MDA-MB-453, MDA-MB-231, and HCC1937, selected based on their different type 1 gene and p53 gene status. These cells were embedded in Matrigel™, BME2rgf, and collagen I with different biochemical conditions (perfusion *vs* static). The cells were exposed to a series of anti-cancer drugs (*paclitaxel, olaparib, cisplatin*) wherein they found different drug responses in comparison to 2D cultures. The HCC1937 triple negative breast cancer cells showed maximum reduction in cellular viability at much lower concentration of *paclitaxel* in 2D culture as compared to 3D. It was also observed that on-chip culture condition improved the overall cell viability due to the constant perfusion of the medium (Fig. 4b) [21]. Hence, a better drug-screening platform was proposed to directly culture patient-derived material, which displayed better drug response as compared to 2D model. As a step forward, a study increased the heterogeneity of the tumor-on-chip model by using four cell types to establish the 3D tumor-on-chip model including cancer cells (Her2+ subtype), CAFs, immune cell, and endothelial cells. Immune cells play a crucial role in tumor progression and drug response while most *in vivo* models are immunocompetent. This study suggested a role of trastuzumab in immune cell mediated toxicity and role of CAFs in antagonizing the effect of *trastuzumab* [142]. Thus, this is delving to bring immune component in an *ex vivo* platform to study drug screening.

Another important feature of tumor progression is angiogenesis, which is challenging to study in 2D based model. Several tumor-on-chip models are reported in investigating the mechanism of angiogenesis and testing the efficacy of anti-cancerous drugs targeting the formation of the new vasculature. As an example, Nashimoto et al., developed a perfusable breast spheroid-on-chip using a tri-culture model (HUVEC, MCF-7, human lung fibroblasts) to show the role of stromal cells in angiogenesis. This study showed the response of drug (*paclitaxel*) in static and perfused condition. In static condition, drug response was dose dependent while in perfused condition, it was independent on drug doses [143]. Thus, this study emphasized the importance of including the vascular network for better drug screening platforms.

Overall, the above-mentioned examples manifest how microfluidics can significantly contribute to evaluate the efficiency and toxicity of drugs, their pharmacokinetics/pharmacodynamics, as well as some aspects of administration, distribution, metabolism, and excretion.

### 6.3 High throughput breast tumor-on-chip for multiple data analysis

Tumor-on-chip models are beneficial for preclinical drug screening, as they are intended to perform high-throughput analysis of anti-tumor drugs and other biological factors. To perform high-throughput analysis, a device should be capable to run many tests in parallel with high level of reproducibility, homogeneity, and high fidelity [144]. As an example, Chen *et al*., reported the design of a microfluidic device to study early metastasis [145]. The device included three hydrogel regions separated by media channels. The fibrin gel and HUVEC suspension were filled in the central channel and the two lateral channels were seeded with human lung fibroblast in fibrin suspension. The device was used to study trans-endothelial migration and pre-metastasis. This device was utilized for higher parameters analysis and rapid quantification of large data. This multi-well invasion chip consisted 4000 microwells, which included square (200 × 200 μm^2^) and round (200 μm diameter) wells, with a depth of 160 μm. The chip was employed for investigating the invasion, cancer cell behavior and high throughput screening of rare samples and drugs.

Multiplexed organ-on-a-chip device is another process for large data set generation. Microfluidics-based multiplexed immunohistochemistry platform was used for the simultaneous detection of multiple biomarkers (ER, HER2, PR and Ki-67) from breast cancer cells and tissues [102,146]. In this line, Fang *et al*., designed a microfluidic chip to separate circulating EpCAM-positive exosomes and HER2-positive exosomes from breast cancer cell line and patient [146]. The expression level of these positive exosomes was almost similar to tumor tissues (Fig. 4 c-d). Through this high-throughput technique, multiple biomarkers can be tested instantaneously with improved sensitivity and specificity, and at an affordable price. The microfluidics platform used high-throughput cell-based screening of cells and a rectangular microarray of trapping barriers to trap them [146]. This device permitted the rapid identification of invasive phenotypes based on biomarker expression and biophysical properties.

### 6.4 Disease modeling by multi-organ-on-chip systems

Despite their advanced capabilities, single organ-on-chip systems cannot recapitulate the interaction between different tissues and organs. This is of paramount importance in drug screening to know the metabolites of drugs can be toxic in other organs. For this reason, organ-on-chip technology is evolved towards the integration of multiple organ function on-chip. Microfluidic multi-organ-on-chip (MOC) systems include multiple tissues or organ models interconnected following a physiological order. MOC models provide multiple advantages to understand the pharmacokinetics/pharmacodynamics of drugs, as well as their metabolism and toxicity. This type of multi-organ models is an advancement towards the development of personalized *in vitro* models in the form of human- or body-on-chip models. They recapitulate better the actual effect of drugs on individual patients. Recently, a multi-organ-on-chip model was reported recapitulating the first-pass drug absorption, metabolism and excretion in humans to analyze drug response and toxicity [8]. The same group also devised two-channel eight organs (intestine, liver, kidney, heart, lung, skin, blood–brain barrier and brain) vascularized system to study the dosing of drugs, drug toxicity in non-targeted/associated organs and reveal the mechanism behind the disease. In cancer therapeutics, a multi-organ-on-chip device was reported which comprise lung, liver and breast cancer tissue. The effect of curcumin on breast cancer cells was examined through inhalation and intravenous delivery [147]. The result indicated that intravenous delivery significantly decreased breast cancer viability compared to inhalation therapy.

To illustrate the trans-endothelial immigration of breast cancer cells within a bone-imitating microenvironment a tri-culture microfluidic system consisting hBM-MSCs lined with endothelium, and MDA-MB-231 breast cancer cells were developed [116]. This study concluded that multi-organ-on-chip model could be utilized to understand breast cancer biology and new therapeutic screening. Another study exhibited that breast cancer cells after trans-endothelial migration into bone microenvironment stimulated the formation of bone cancer. This led to activation of the ERK1/2-RUNX2 signaling pathways in cancer cells that provided drug resistance [148].

Thus, the multi-organ-on-chip model can be useful to understand the links between mechanobiological aspects and the development of better anti cancerous therapeutics.

## 7 Industrial progress in microfluidics-based point-of-care diagnostic devices

Since the early 90´s, the microfluidics field is progressing rapidly. In 2018, the worldwide market size was estimated at approximately USD 10.06 billion and it is expected to cross USD 27.91 billion by 2023 [163]. Well-known pharmaceutical and biotechnological companies, such as Abbott, Ibidi, or Roche Diagnostics and among others, are using microfluidics as point of care diagnostic devices, gene therapy and editing, or for modeling *in vitro* biochemical and biophysical features of cells or tissues. Similarly, during the last years, small and medium-sized enterprises from all over the world, such as Elvesys, Dolomite, Fluigent, Darwin, BlackholeLab, or Micronit, among others, have emerged focusing their effort in the development of innovative microfluidics technologies for biomedical applications.

The global microfluidic device market is expected to gain impetus from increasing technological advancements in the life sciences and biomedical domain. The microfluidic market is segmented into diagnostic centers, research institutes, hospital, pharmaceutical and biotechnology companies, healthcare facilities and others. Additionally, according to a recent report [164], the global microfluidic market is also segmented based on device type, materials and applications. The chip segment is the most dominating due to their great demand in various applications, such as biomedical, drug delivery, immunoassay and other biotechnological applications. In addition, the chip segment is expected to be dominant in the near future because of its cost effectiveness and easy to use. By application segments, pharmaceuticals and life science researches are rapidly adopting microfluidics for their research in drug development and screening, early detection, and disease diagnosis.

In breast cancer research, many companies are utilizing microfluidic devices for the early detection and diagnosis of the disease. As mentioned above, such devices are used for the rapid, efficient, and automatized screening of anti-cancer drugs. In this direction, BioIVT's Elevating Science Industry (USA) has developed oncology Tissue Microarrays (TMAs), which can be applied as a screening tool for multiple cancer patient tissue samples. TMAs are compatible with immunohistochemistry and *in situ* hybridization. These TMAs for several cancers (breast, prostate, lung and colorectal) can be applied to discover new proteins or genetic markers and provide efficient technology for disease diagnosis and validation. Mimetas™ (The Netherlands) has advanced a high throughput OrganoPlate® platform to culture breast cancer cells and tissue embedded in a 3D hydrogel. This microfluidic platform allows the instantaneous culture of 96 micro-tissues by perfusing limited amounts of culture medium or growth factors, which is paramount importance for drug screening of patient-derived materials. OrganoPlate® Graft is the first *in vitro* cell culture platform developed by Mimetas™ that permits spheroids, organoids, and tumors to develop vasculature. This is useful for drug administration. This technology can be used in personalized medicine for appropriate drug selection and therapy.

InSphero (USA) has set the standard for *in vitro* drug testing. They have designed 3D InSight™ Microtissues, which have a ready to use *in vitro* 3D organotypic cell culture model to evaluate drug efficiency and toxicity. InSphero has also developed a customized tumor model using a 3D Select™ system by optimizing cell composition, intercellular interaction, tissue structure, and biological characteristics of primary tumors. It is amenable to a variety of biochemical and phenotypic for microtissue formation to develop monoculture and co-culture model. InSphero has designed the 3D InSight™ Microtissues model for the drug toxicity assessment of lung, kidney, liver, pancreas, ovary and breast cancers.

**Fig. 6:**
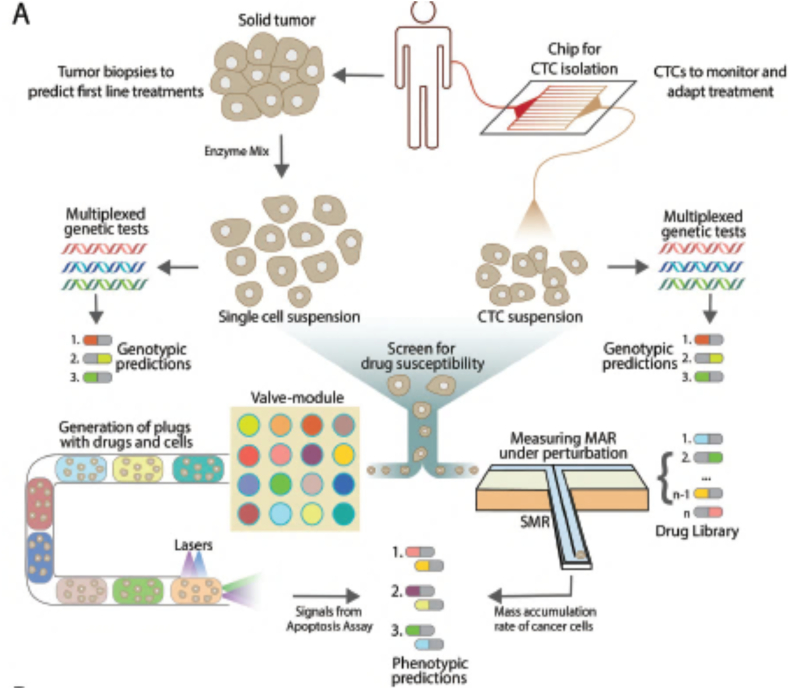
Overview of the functional and genetic cancer patient stratification and disease diagnosis approach using microfluidics. Tumor biopsies can be used to determine first line of treatment strategy based on their genetic and phenotypic data. The droplet-based microfluidic technology can be used to isolate CTCs from patients to monitor the disease state. The mass accumulation rate can be monitored through a series of suspended microchannel resonators to allow drug susceptibilities of patient derived tumor cells or CTCs. In combination, genotypic testing provides the information to predict the effect of potent drugs from patients' genotypes. These data can be used by clinicians for the selection of drug for precise treatment therapy. Reproduced with permission from ref. ^[^^151^^]^ (Creative Commons Attribution 4.0 International License (CC BY 4.0)).

μFluidics (USA) has developed microfluidic point-of-care chips, which is highly sensitive for the rapid detection of biomarkers from various diseases, such as cancer. These chips can easily be automated and can be integrated with nucleic acid amplification chips to capture disease biomarkers and to detect by on-chip nucleic acid amplification methods. Scienion (Germany) is expanding a microfluidic component for multi-parametric miRNA analysis from a variety of cancer (breast, lung, thyroid, pancreatic, and liver). This array-based approach for multi-parameter detection shows high efficiency, effectivity, and a reduced time and sample consumption. IDEX Health and Science (USA) has evolved a silicon nanowire-based biosensor device for the detection of tumor biomarkers (protein and circulatory tumor DNA) from liquid biopsies. Ibidi (Germany) is manufacturing a variety of microdevices, which can be used to study cancer cells behavior, metastasis and chemotaxis. Emulate™ (USA) is also working on organ-on-chip technology and producing supporting instruments to reduce the complexity of chip development. The company is working on the development of a chip to understand diseases complexity, drug development and working towards the development of personalized medicine. Thus, the microdevices fabricated by these industries can restructure the organ and tissue by providing the entire physiological and mechanical stimulus like body condition through pressure sensor device. These miniaturized devices are used to isolate and identify tumor biomarkers present on single cells. They can also be utilized for genetic and phenotypic data analysis of cancer cell patients for future personalized cancer treatment therapy (Fig. 6). Overall, the microfluidics industry has boosted their portfolio of products and applications, which will influence the pharmacological and clinical market in the near future.

## 8 Summary and future perspectives

Microfluidics technology can significantly contribute in boosting the understanding of cancer pathophysiology and drug screening for personalized medicine. This technology combines the culture of human cells with microfabricated devices capable to control different parameters within the chip, such as interstitial pressure, gradient generation, or vascularization. This results in the development of well-defined and physiologically relevant 3D tumor models, which can be employed for the rapid screening of therapeutic compounds with greater accuracy and eventually, may replace animal testing in near future.

Microfluidics allow the investigation of complex biological fluids in a simple manner and offers non-invasive approaches for the early detection and diagnosis of cancer. There are different breast cancer specific biomarkers in the blood, such as DNA, miRNA, proteins, hormones, exosomes and CTCs. However, these biomarkers are present in high concentrations in tumor and eventually are diluted in the plasma, which is difficult to detect by conventional analytical assays (the targeted protein amount with respect the background ratio is approximately 1:10^5^, which is beyond the detection limit). Microfluidics are progressively addressing the challenges related to sample volume, cost effectiveness, and others. This system provides a high throughput sample screening at greater sensitivity and accuracy.

Emerging microfluidic technologies in (breast) cancer research include droplet-based microfluidics devices. These systems are used in breast cancer for hormonal screening (such as estrogen, progesterone) from the tissue samples. The sample volume for droplet microfluidics is less than 1000 times than the currently used methods. In this way, a smaller volume of samples is employed to monitor the anti-estrogen treatment strategy. The ability to measure routinely the estrogen/progesterone level after antitumor treatment will give a new way to monitor the anti-cancerous drug response and provide a way for drug development.

The microfluidics technology in combination with immunoaffinity, Raman Scattering and ultracentrifugation is helpful for the detection of a large data set generated from these biomarkers present in liquid biopsy, blood plasma, and breast milk. The microfluidics-based multiplexed technology also utilizes to monitor the therapeutic based biomarkers (surface modified with anti-CD9, anti-CD63, antitumor drug) response in cancer diagnosis and classification measure [149]. In this way, the microfluidics technique provides automated, fast and high-quality quantitative *in situ* biomarker data at low-cost.

This microfluidic field also throw the light to understand the impact of CTCs into distant organ metastasis through multi-organ-on-chip technology. This technology can be utilized to monitor the functionality and inter tissue interactions. Bone and liver are more affected with breast cancer metastasis. Their interactions can be monitored by growing the cells from the tissue/organ of breast cancer, bone, and liver, which can be connected through flexible microchannels (bionic blood vessel) to achieve multi organ integration [8]. This multi organ-on-chip will be an advantageous tool to study the interactions between the stromal and breast tissue. The chemical secreted from these stromal tissue helps in tumor progression. In this way, it can be helpful to identify the new pathway in breast cancer metastasis, disease modeling and for drug delivery applications.

The integration of automated real-time image monitoring and screening into breast tumor-on-chip device may be helpful to monitor the tumor growth and progression. Although it is challenging and difficult to achieve with standard methodologies. However, the extracted biopsy only represents a single snapshot at a time and the selected specimen may not represent the actual tumor heterogeneity and provide inadequate information. Through automated imaging system, multiple or sequential biopsies at a single time may provide valuable information about tumor development. In combination with automated optical imaging techniques into breast tumor on-chip systems together with data interpretation tool is expected to provide physicians and pharmaceutical industries with a new hope for understanding the cancer development and throw a light on the possible therapeutic approach [150].

Finally, it is worth noting that microfluidics still faces some challenges, which need to be solved before being adopted by the clinics. For example, their fabrication is still complicated and requires expertise and skills in nanotechnology, suitable biomaterials, and sophisticated tissue engineering techniques for developing relevant vascularized architectures that are reminiscent of tumor pathophysiology. For the particular case of breast tumor-on-a-chip models, the integration of different cell types and tissues of typical breast metastatic sites with different media requirements is a still a limitation that needs to be addressed. Further, the limited availability of the primary cells and tissue samples from breast cancer patient is another hurdle in this field. The use of human iPSCs holds very promising for the generation of relevant on-chip models. Next, the use of xeno-free biomaterials would help to recreate better the biochemical and structural complexity of the native 3D microenvironment. From a technological perspective, it is difficult to conduct multiple processes, such as biomarker separation, detection, analysis, and retrieval of information from a single chip. In addition, further refinement to current models may include the generation of precise gradient flows and shear stresses as relevant mechanochemical cues that are required for endothelial cells and breast cancer cells, reproducing the *in vivo* conditions. Overall, despite the current technological and biological limitations, breast tumor-on-chip models are gaining attention due to its ease of use, efficiency, and relevance for the detection of breast tumor biomarkers from patient samples.

The multi-organ-chip system looks impressive and gives a hope to generate body-on-chip. This still requires maintenance of stable fluid connection, avoid bacterial contamination and monitor cell viability throughout the culture process. As the number of organs on the chip increases, the complexity of the system is enhanced, which may give unpredictable results. The microfluidic Industries are proceeding in this direction to solve some of the problems related to biological complexities in consultation with medical scientists. These Industries regularly improve their technologies, which can help to predict the disease development at early stage and optimize the drug profiling using single and pairwise standard drug combinations. Thus, this miniaturized technology can be used in multiple putative applications in biomedical, pharmaceutical and biotechnological systems for drug discovery and development.

## 9 Conclusions

Organ-on-chip technology is progressively emerging as an *in vitro* platform to grow human-like tissue/organ. In this respect, tumor-on-chip model can compensate the use of 2D and 3D models by recapitulating 3D breast tumor microenvironment, co-culturing breast tumor cells along with neighboring stromal cells and create a more physiologically relevant tumor model. This organ-on-chip model can also fulfill the criteria to study metastasis cascade and perform *in vitro* drug screening platform. Advancement in this technology can reduce the cost and time of the drug development procedure and increase the precision of breast cancer therapy. This technology will be helpful for the identification and development of new cancer biomarker/therapeutic in breast cancer applications. In the long term, this technology may pave the way to develop personalized drug screening platform based on breast cancer biomarkers present in patient derived breast tumor.

## Declaration of Competing Interest

SB, JA and GVC are employers of Elvesys. Elveflow is an Elvesys brand. Authors declare no other conflicts of interest.
